# Application of a novel hybrid algorithm of Bayesian network in the study of hyperlipidemia related factors: a cross-sectional study

**DOI:** 10.1186/s12889-021-11412-5

**Published:** 2021-07-12

**Authors:** Xuchun Wang, Jinhua Pan, Zeping Ren, Mengmeng Zhai, Zhuang Zhang, Hao Ren, Weimei Song, Yuling He, Chenglian Li, Xiaojuan Yang, Meichen Li, Dichen Quan, Limin Chen, Lixia Qiu

**Affiliations:** 1 Department of Health Statistics, School of Public Health, Shanxi Medical University, Taiyuan, 030001 Shanxi China; 2 Key Laboratory of Public Health Safety of Ministry of Education, School of Public Health, Fudan University, Shanghai, 200032 China; 3 Shanxi Centre for Disease Control and Prevention, Taiyuan, 030012 Shanxi China; 4 Shanxi Provincial People’s Hospital, Taiyuan city, Shanxi Province China

**Keywords:** Hyperlipidemia, Bayesian network, Hybrid algorithm, Inter. Iamb-Tabu

## Abstract

**Background:**

This article aims to understand the prevalence of hyperlipidemia and its related factors in Shanxi Province. On the basis of multivariate Logistic regression analysis to find out the influencing factors closely related to hyperlipidemia, the complex network connection between various variables was presented through Bayesian networks(BNs).

**Methods:**

Logistic regression was used to screen for hyperlipidemia-related variables, and then the complex network connection between various variables was presented through BNs. Since some drawbacks stand out in the Max-Min Hill-Climbing (MMHC) hybrid algorithm, extra hybrid algorithms are proposed to construct the BN structure: MMPC-Tabu, Fast.iamb-Tabu and Inter.iamb-Tabu. To assess their performance, we made a comparison between these three hybrid algorithms with the widely used MMHC hybrid algorithm on randomly generated datasets. Afterwards, the optimized BN was determined to explore to study related factors for hyperlipidemia. We also make a comparison between the BN model with logistic regression model.

**Results:**

The BN constructed by Inter.iamb-Tabu hybrid algorithm had the best fitting degree to the benchmark networks, and was used to construct the BN model of hyperlipidemia. Multivariate logistic regression analysis suggested that gender, smoking, central obesity, daily average salt intake, daily average oil intake, diabetes mellitus, hypertension and physical activity were associated with hyperlipidemia. BNs model of hyperlipidemia further showed that gender, BMI, and physical activity were directly related to the occurrence of hyperlipidemia, hyperlipidemia was directly related to the occurrence of diabetes mellitus and hypertension; the average daily salt intake, daily average oil consumption, smoking, and central obesity were indirectly related to hyperlipidemia.

**Conclusions:**

The BN of hyperlipidemia constructed by the Inter.iamb-Tabu hybrid algorithm is more reasonable, and allows for the overall linking effect between factors and diseases, revealing the direct and indirect factors associated with hyperlipidemia and correlation between related variables, which can provide a new approach to the study of chronic diseases and their associated factors.

**Supplementary Information:**

The online version contains supplementary material available at 10.1186/s12889-021-11412-5.

## Introduction

Cardiovascular and cerebrovascular disease (CVD) is the leading disease that threatens human health worldwide and has become one of the leading causes of death [1, 2]. Atherosclerosis is the main cause of CVD. Hyperlipidemia, as the most important risk factor for atherosclerosis, plays an important role in the occurrence and development of CVD [3, 4]. However, with China’s economy picking up pace, dietary structure changing, pace of social life accelerated, the detection rate of hyperlipidemia is on the rise [2, 5, 6]. Hyperlipidemia has become an important public health problem, and according to existing studies, prevention and control of hyperlipidemia can play a significant role in the first- and second-degree prevention of cardiovascular disease [7, 8]. Therefore, it is particularly important to comprehensively analyze the related factors of hyperlipidemia and the complex relationship between these factors to prevent the occurrence of hyperlipidemia.

In the past, most of the research on the related factors of hyperlipidemia was based on logistic regression analysis with independent variables. In fact, the assumption of variable independence in logistic regression is difficult to achieve. In addition, logistic regression cannot distinguish direct and indirect related factors of hyperlipidemia. In the study of medical biological effects, there may be complex network structure relationships between diseases and factors, and between one factor and another factor. This relationship may be an overall linkage effect, that is, a change in the controllable link will lead to a change in the overall effect. The results of logistic regression analysis do not reflect this overall linkage effect. In contrast, the BNs proposed by Pearl Judea for the first time in 1987 has no strict requirements on statistical assumptions [9]. By constructing a directed acyclic graph(DAG) to reflect the potential relationship between variables, the conditional probability distribution table is used to reflect the correlation strength, which can intuitively describe the complex cyber risk mechanism between disease and factors [10], overcoming some of the deficiencies of logistic regression [11, 12]. In addition, when conducting risk reasoning, BNs can reason the probability of unknown node according to the state of a known node, and can more flexibly determine the risk of dyslipidemia [13–15].

The creation of BNs can be divided into two categories: Structure learning and parameter learning [16]. The focus of this paper is to study the algorithm of BN structure learning. Parameter learning will directly use maximum likelihood estimation. The structure learning algorithm mainly includes the constraint-based [17, 18] and the score-and-search-based approach [19]. The former, which enjoys high learning efficiency, can obtain the global optimal solution. However, with the increase of the number of variables, the number of conditional independence tests between variables will increase exponentially. Also, the results of high-order conditional independence test are not reliable. Although the latter allows for a more accurate network structure, searching for the optimal BNs structure from all possible network structures proves an nondeterministic polynomial time (NP)-hard problem for huge search space. Given these limitations of the two types of algorithms, some researchers have proposed the hybrid algorithms that accumulate both score-and-search-based and constraint-based approaches of structure learning [20]. Firstly, the undirected network framework is builted by the constraint-based methed to reduce the size of the search space, and then the search score method is used to determine the direction of the edge in the network to find the optimal network structure. One of the most widely-used hybrid algorithm represents the Max-Min Hill-Climbing (MMHC) algorithm [21]. Firstly, the constraint-based approach Max-Min parents and children (MMPC) is used to infers a skeleton of BN, and then uses a Bayesian scoring Hill-Climbing search to determine the orientations of the edges in the skeleton. However, the Hill-climbing method is a local optimal algorithm [21], and the MMPC algorithm has more inspections in the first-stage conditional independence test, which is likely to cause inaccurate test results. As such, this study attempts to improve the MMHC with some algorithms with better performance.

The idea of improving the hybrid algorithm MMHC is as follows: Firstly, select some current better performance methods based on conditional independence testing to replace the MMPC algorithm, such as inter.iamb [22] and fast.iamb [23]. The operation results of the above two improved algorithms have been improved, and they are currently relatively satisfactory algorithms based on conditional independence testing. Secondly, use the global tabu search algorithm based on the scored search to determine the direction of the edge. Tabu search is a metaheuristic approach proposed by Glover and it is one of the most efficient optimization techniques that incorporates adaptive memory to escape local search and find the global optimum [24]. In 2015, Xuelei Zhang confirmed that the optimization effect of Tabu algorithm is better than Hill climbing and K2 [25], and in the later period, other members of the research group also applied the Tabu algorithm to the network construction of other diseases in the medical field, and all achieved good results [26–28]. Finally, three new hybrid algorithms are obtained, such as MMPC-Tabu, Inter.iamb-Tabu, and Fast.iamb-Tabu. We perform a performance comparison with widely used MMHC hybrid algorithm on randomly generated datasets to assess their performance. And then the optimized BN was applied for exploring the related factors of hyperlipidemia.

In this study, we aim to explore a novel hybrid algorithm of BN to portray the inherent relationships between hyperlipidemia and its associated factors, to predict the risk of hyperlipidemia in BNs model, to compare the effects of Logistic regression model and BN model on results interpretation and risk reasoning, and to provide a new model construction method for the study of factors affecting hyperlipidemia.

## Materials and methods

### Simulating datasets

**Fig. 1:**
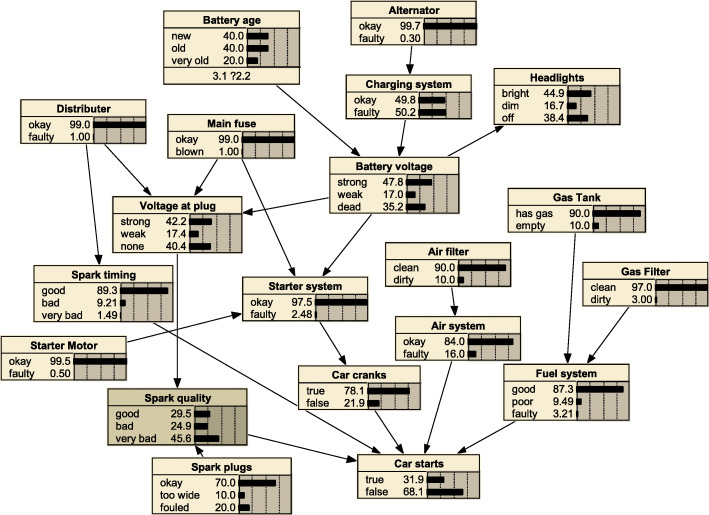
Car Diagnosis network structure

The Car Diagnosis model model was selected using Netica software to generate randomly simulated data with different sample sizes. To increase the comparability of the algorithm, each sample size of the simulation data set was generated 10 times repeatedly. The Car Diagnosis network is a network of relationships between various parts of a car and vehicle maintenance. It consists of 20 nodes with 22 directed edges, as shown in Fig. 1.

### Instance dataset

#### Study design and participants

The design of this study is a partial cross-sectional survey conducted by the “China Adult Chronic Disease and Nutrition Surveillance (2015)” project in Shanxi Province. Participants were enrolled in the China Adult Chronic Disease and Nutrition Surveillance conducted in Shanxi Province in 2015. The survey adopted a multi-stage stratified random sampling method for a representative sample. In the first stage, eight representative monitoring points such as Datong Xian, Shuocheng Qu, Lin Xian, Xinghua Ling, Pingding Xian, Yuci Qu, Huguan Xian, Jiang Xian were randomly selected in Shanxi Province. These monitoring points are relatively evenly distributed in Shanxi Province. In the second stage, three townships (streets, groups) were randomly selected using a systematic sampling method sorted by population size from each monitoring point. In the third stage, the same method was used to randomly select 2 administrative villages (residential committee, organizations) within the selected township (street, regiment). The fourth stage involved random selection of one group containing 60 and above households from each selected village/residential committee/organization. In the five stage, 20 households were selected for the dietary survey and 25 households for the non-dietary survey, for a total of 45 households from each sampled group. In the final stage, chronic disease and nutrition personal questionnaires were administered to all permanent residents aged ≥18 years in each household. Finally, a total of 4845 participants completed the survey and physical examination. This study was approved by the China Chronic Disease Center Ethics Committee, with reference number 201519. Informed consent was signed by all study participants or their agents. All experiments were performed according to the relevant guidelines and regulations.

The inclusion criteria for this study are all residents who are 18 years of age or older and have lived in the monitoring area for more than 6 months in the past 12 months. Exclusion criteria for this study were those residents who lived in functional areas, such as sheds, military or student dormitories, nursing homes, and so on. (An additional word file shows the data collection and definitions in more detail [see Additional file 1]).

### Bayesian network

The BNs was first proposed by Pearl Judea in 1987 and has since been widely used (25). It is essentially a joint probability distribution that describes the probability of dependencies between variables. Given a series of random variables *X = { X*_1_, ⋯, *X*_*n*_ *}*, then its joint probability *P(X*_1_*,* ⋯, *X*_*n*_*)* can be expressed as a BNs *B = (G, θ)*, in which G is structure which demonstrates the random variables and the relations between them by a DAG. In a DAG, random variables and relations are nodes and edges, respectively. θ is a set of local parameters that denotes the conditional probability distributions for the values of each variable according to the structure G. These measures are saved as a table for each variable which is called conditional probability table (CPT) [29].

### The constraint-based approach

#### MMPC algorithm

The MMPC algorithm [30] uses a two-stage scheme to obtain candidate parents and children (CPC) for each variable. In phase I, the variables are sequentially entered into the CPC by using a heuristic function, and the first stage ends if all remaining nodes are independent of the target node T. In phase II, MMPC attempts to remove the false positives that may have entered in the first phase. This is achieved by testing whether *Ind(X; T|S)* for some subset *S ⊆ CPC*. If the condition holds, X is removed from *CPC* [21]. When the MMPC algorithm is used in the first-stage conditional independence test, the number of tests is large, which may easily lead to inaccurate test results.

#### Inter.Iamb algorithm

The Interleaved Iamb (Inter.iamb) algorithm [22] is an evolutionary algorithm of the iamb algorithm. The Incremental association Markov blanket (Iamb) algorithm [22] consists of two phases, a growing phase, and a shrinking phase. In the growing phase, Candidate Markov blanket (CMB) collection starts with an empty sequence and uses the heuristic search function *f(X; T|CMB)* to evaluate variables that are not yet in the CMB, i.e. to evaluate the degree of association between the variable X and the variable T under the condition of giving CMB. Entry into the CMB is determined by determining whether the value of the variable with the largest value of the heuristic search function is greater than a given threshold until the CMB no longer changes. In the shrinking phase, variables that are not part of the MB(T) are removed one by one by checking whether the variable X in the CMB is independent of T in the given variables in the CMB. The Inter.iamb algorithm adds the second stage of the independence test process in the first phase of iamb, which reduces the false positive relationship in the CMB, thereby reducing the number of conditional tests and making the measure of relevance more accurate.

#### Fast.Iamb algorithm

The Fast.iamb algorithm [23] is also an algorithm optimized for iamb, enabling it to find the MB(T) of the target variable T faster. Similar to iamb and Inter. iamb, Fast.iamb contains a growing phase followed by a shrinking phase. The difference is that in each loop of the Growing stage, it sorts each variable from big to small according to the heuristic search function *h* value, and then adds the candidate variable to MB(T) according to the size of the dependency. Its heuristic search function *h* uses the more statistically appropriate significance of a G2 conditional statistical test, rather than the raw conditional information value, as iamb and Inter-iamb do. The core idea of Fast. Iamb algorithm is to reduce the number of conditional independence tests by adding multiple candidate variables to MB(T) after reordering variables, so as to improve the speed of markov blanket. 

### The score-and-search-based approach

#### Tabu algorithm

The Tabu Search Algorithm [31] is a sub-heuristic algorithm for simulating human memory functions. It is characterised by few parameters, simple structure and strong global optimisation capability. For a given current network structure, under the premise that does not produce network loop, the algorithm uses three operations namely edge addition, subtraction edge and reverse edge to generate neighborhoods. Then, search for the local optimal solution in the neighborhood, put it into the taboo table, and record the local optimal solution that has been searched through the taboo table. Consequently that the optimal solution can be avoided as far as possible in the next search and the cycle repetition of the search process can be avoided. Cooperate with the contempt criterion to “pardon” some of the optimal solutions in the tabu table and ignore the tabu restrictions. These two steps are iterated and the taboo table is continuously updated to obtain the global optimal solution. The local optimisation of the hill climbing algorithm is compensated for.

#### Hill-climbing algorithm

The goal of the Hill-Climbing Algorithm [32] is to find the model with the highest score. It starts with an initial model, which is usually set as an unbounded model. In each step of the search, the current model is first modified locally by three search operators: edge addition, subtraction edge, and transition edge, to obtain a series of candidate models; then calculate the score of each candidate model and compare the optimal candidate model with the current model; if the optimal candidate model has a higher score, continue the search with it as the next current model; otherwise, the search is stopped and the current model is returned.. Because of the initial network selection, the hill climbing method is easy to fall into local optimum and cannot find the global optimal network.

### Hybrid algorithm

In the first stage, the algorithm of conditional independence testing is used to determine the edges of the network and construct the network framework. The second stage uses a global tabu search algorithm based on score search to determine the direction of the edge.

### The analysis method of related factors of hyperlipidemia

First, we employed the histogram, box plot and composition comparison to describe the basic characteristics of the survey population, and to describe the detection rate of hyperlipidemia for understanding of its distributions. Second, we used Chi-square test to compare the detection rate of hyperlipidemia among people with different characteristics. *P values* less than 0.05 were considered to be significant, and all tests were two-sided. Afterwards, stepwise logistic regression was used for screening of the variables to determine the main factors, with *α*_*(in)*_ = 0.05 and *α*_*(out)*_ = 0.10. Variables with a *p value* of less than 0.50 on univariate analysis were considered candidate variables for a stepwise logistic regression model. Also, the model was used for risk prediction. We then used the initial screening variables, and the optimal hybrid algorithm to construct the BN of hyperlipidemia. Last, the maximum likelihood estimation is used to calculate the conditional probability between the child node and the parents node. The possibility of hyperlipidemia was studied by model reasoning, and the effects of the Logistic regression model and BN model on result interpretation and risk reasoning were compared.

#### Chi-square test

The chi-square test is the degree of deviation between the actual observation value of the statistical sample and the theoretical inferred value. The degree of deviation between the actual observation value and the theoretically inferred value determines the size of the chi-square value. The larger the chi-square value, the less consistent; the smaller the chi-square value, the smaller the deviation, and the more consistent it is. If the two values are completely equal, the chi-square value is 0, indicating that the theoretical value is in complete agreement. The chi-square test can be employed for evaluation of a relationship between two categorical variables.

#### Stepwise logistic regression

Stepwise logistic regression model, which comprises automatically selecting a reduced number of predictor variables, aims to establish the most outstanding logistic regression model. Its principle is to first introduce all models to construct a regression model, and then use the screening criteria to rank all variables, and eliminate the independent variable with the least correlation with the dependent variable from the model.

### Statistical analysis

Randomly generate simulation data sets of different sample sizes using Netica(Norsys Software Corp., Vancouver, BC, Canada); the BN model of simulated data was established by using package bnlearn() in R program 3.6.1 (R core team).

Statistical description, univariate chi-square test, and multivariate logistic regression were conducted in IBM SPSS Version 22 (IBM Corp., Armonk, NY, USA). After that, the screened factors were included into BNs modeling, which was run in “bnlearn” packages of R program 3.6.1(R core team). R-codes details see Additional file 3. We set inter.iamb-Tabu hybrid algorithm to establish the BNs structure, and the maximum likelihood method to acquire the parameters for conditional probability distribution respectively. The BN graph and BN inference model were drawn by Netica(Norsys Software Corp, Vancouver, BC, Canada).

## Results

### Networks performance evaluation of three hybrid algorithms

We tested our algorithm with datasets generated from Car Diagnosis benchmark network. The BNs were inferred by the MMPC-tabu, Inter.iamb-tabu, Fast.iamb-tabu hybrid algorithms and MMHC algorithm respectively. Using the MMHC algorithm as a comparison, compare the inferred networks with the benchmark networks, the more similar the inferred networks are to the benchmark networks, the better the hybrid algorithm. The similarity was expressed by calculating the number of reverse edges R(E), the number of missing edges M(E), the number of redundant edges A(E), and their sum S(E). When calculating S(E), the weight of the reverse side is set to 0.5. In order to increase comparability, this study compares the average results of each algorithm after 10 runs, and the smaller the value of each evaluation criterion, the more similar the inferred networks are to the benchmark networks.

#### Car diagnosis network construction effect

Table 1 show the learning effects of the four algorithms on the Car diagnosis network under different sample sizes. It can also be seen that there are few extra edges and the missing side phenomenon was serious, indicating that the possibility of network overfitting is small, and missing edges are the main problem. As the sample size increases, the phenomenon of missing edges can be significantly improved, the constructed BNs are gradually approaching the benchmark network. Under different sample sizes, the modeling effect of the Inter.iamb-tabu hybrid algorithm was better than the other three hybrid algorithms, the total number of errors decreased from 16.0 when the sample size was 100 to 6.9 when the sample size was 20,000, and while finding more edges, it also reduced the situation of reverse edges, the modeling effect was significantly improved. The modeling effect of the Fast.iamb-tabu hybrid algorithm was second only to Inter.iamb-tabu when the sample size was 5000 and above, and the MMPC-tabu hybrid algorithm was slightly better than the MMHC algorithm. It shows that the Inter.iamb-tabu hybrid algorithm can be effectively used in BN structure learning.

**Table 1:** Hybrid algorithm construction of different sample sizes Car diagnosis Bayesian network modeling effect comparison

Sample size	algorithm	R(E)	M(E)	A(E)	S(E)
100	MMHC	3.2	14.2	0.2	16.0
	MMPC-Tabu	3.2	14.2	0.2	16.0
	Fast.iamb-tabu	2.6	16.4	0.2	17.9
	Inter.iamb-tabu	3.2	14.2	0.2	16.0
500	MMHC	4.2	11.7	0	13.8
	MMPC-Tabu	4.2	11.7	0	13.8
	Fast.iamb-tabu	3.3	12.5	0	14.15
	Inter.iamb-tabu	4.6	11.3	0	13.6
1000	MMHC	4.6	10.5	0	12.8
	MMPC-Tabu	4.6	10.5	0	12.8
	Fast.iamb-tabu	3.6	11.5	0	13.3
	Inter.iamb-tabu	5.1	10.0	0	12.55
5000	MMHC	5.2	9.8	0	12.2
	MMPC-Tabu	4.7	9.6	0.1	12.05
	Fast.iamb-tabu	5.2	8.8	0.1	11.5
	Inter.iamb-tabu	6.1	7.4	0	10.45
10,000	MMHC	7	8.2	0	11.7
	MMPC-Tabu	5.7	8.1	0	10.95
	Fast.iamb-tabu	5.3	6.9	0	9.55
	Inter.iamb-tabu	5.4	5.4	0	8.1
20,000	MMHC	7.9	7.4	0	11.35
	MMPC-Tabu	6.7	7.1	0	10.45
	Fast.iamb-tabu	5.5	7.5	0	10.25
	Inter.iamb-tabu	5.0	4.4	0	6.9

### Study on related factors of hyperlipidemia in Shanxi province

#### Basic characteristics of the population

**Fig. 2:**
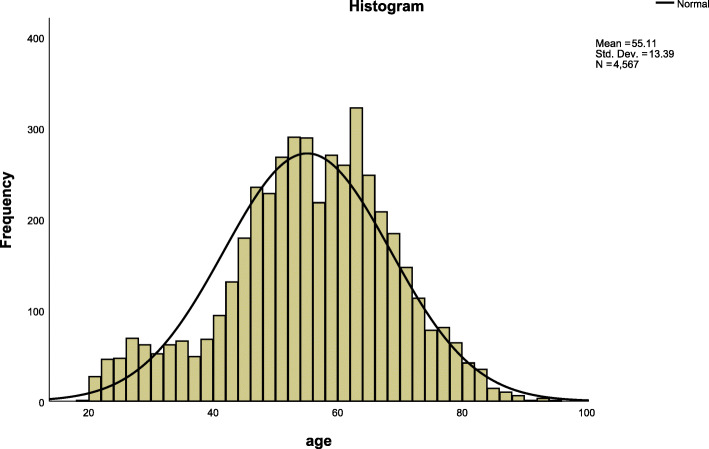
Age distribution of 4567 subjects

**Fig. 3:**
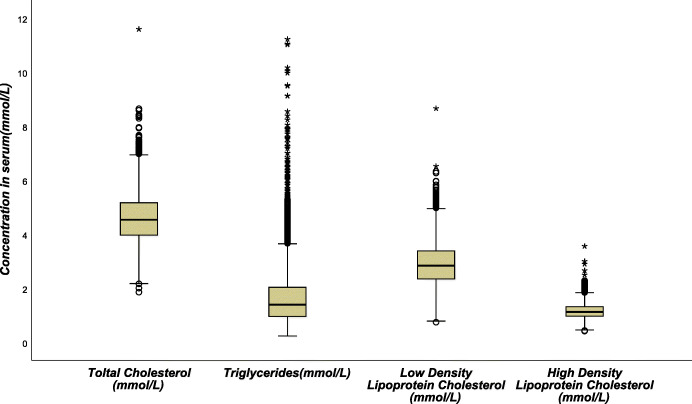
Concentration levels and distribution of four blood lipids

A total of 4567 complete data were left, including 2236 males, accounting for 49.0%, and 2331 females, accounting for 51.0%. The youngest was 19 years old and the oldest was 94 years old, the median age was 56 years old. The age distribution was shown in Fig. 2. The detection rate of hyperlipidemia was 46.3%, and the 95% confidence interval was [44.8, 47.7%]. All four lipid profiles were positively skewed (Fig. 3.). The abnormal rates of TC, TG, LDL-C, and HDL-C were 5.3, 20.0, 7.4, and 34.9%, respectively. Dyslipidemia in Shanxi Province was mainly due to the decrease of HDL-C, followed by the increase of TG.

#### Level of hyperlipidemia in different populations

Supplementary Tables S1–S3 (Additional file 2: Tables S1–S3) show the comparison of the prevalence of hyperlipidemia among subjects with different characteristics. Factors such as gender, smoking, occasional drinkers, insufficient physical activity, higher BMI index, central obesity, hypertension and diabetes mellitus showed a higher prevalence of hyperlipidemia (all, *P-value* < 0.05). Age, marital status, education level, average daily salt intake, and oil intake were not associated with the detection rate of hyperlipidemia.

#### Logistic regression analysis of factors related to hyperlipidemia

We conducted a multivariate logistic regression analysis using a stepwise method(with an entry probability of 0.05, and removal probability of 0.10) to select variables, with the presence of hyperlipidemia as the dependent variable; independent variables were those that were associated with hyperlipidemia in univariate analysis(*P-value*<0.5). The results revealed that hyperlipidemia was significantly associated with gender, smoking, physical activity, daily average salt intake, daily average oil intake, BMI, central obesity, hypertension and diabetes mellitus; central obesity was strongly associated with hyperlipidemia, followed by diabetes mellitus (Table 2). The model, while explaining the extent to which various influences affected the detection of hyperlipidaemia, could not distinguish which variables were directly and indirectly associated with hyperlipidaemia. The variable assignment was shown in the Table S4(Additional file 2: Table S4).

**Table 2:** Logistic regression analysis results

Factors	$\overset{\frown }{\beta }$	*SE*	*wald* χ^2^	*P-value*	*OR*	*OR*(95%*CI*)	
						*Lower*	*Upper*
Gender(x_1_)	−0.346	0.077	20.251	<0.001	0.707	0.608	0.822
Smoking(x_3_)	0.177	0.084	4.429	0.035	1.193	1.012	1.407
physical activity(x_5_)	−0.090	0.045	4.008	0.045	0.914	0.837	0.998
daily average salt intake(x_6_)	0.318	0.145	4.810	0.028	1.375	1.034	1.827
daily average oil intake(x_7_)	−0.357	0.088	16.524	<0.001	0.700	0.589	0.831
BMI(x_8_)	0.417	0.048	75.617	<0.001	1.517	1.381	1.666
Central obesity(x_9_)	0.501	0.075	44.009	<0.001	1.650	1.423	1.913
Hypertension(x_10_)	0.173	0.064	7.390	0.007	1.189	1.050	1.347
Diabetes mellitus(x_11_)	0.442	0.107	17.078	<0.001	1.556	1.262	1.920
Constant	−0.999	0.347	8.266	0.004	0.368		

#### Bayesian network model of hyperlipidemia

**Fig. 4:**
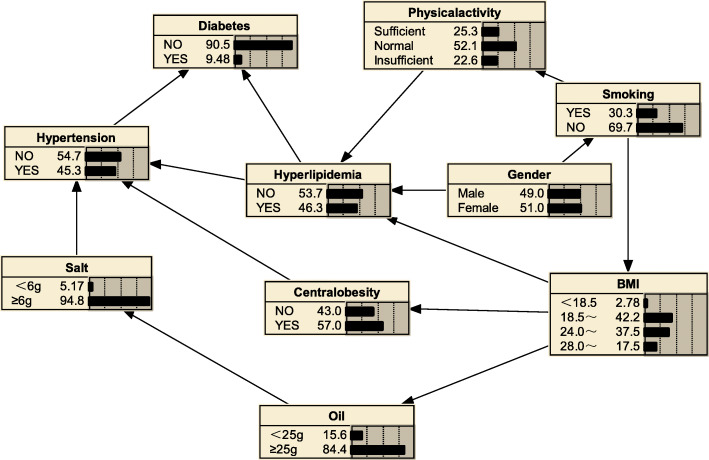
Hyperlipidemia Bayesian network model

According to the nine variables related to hyperlipidemia screened by the previous Logistic regression, the BNs model of hyperlipidemia related factors was constructed by inter.iamb-tabu algorithm, and the maximum likelihood method was used to estimate the probability of each node according to the proposed BNs structure. Figure 4 was the hyperlipidemia probabilistic model established by BNs. It consisted of 10 nodes and 14 arcs. Each node represented one variable, and the arc between connected nodes indicated the probabilistic dependencies. The figures in the nodes indicate the prior probabilities of each node. For example, the prior probability of hyperlipidemia was *P*_*(hyperlipidemia)*_ = 0.463. Gender, physical activity, central obesity, hypertension, and diabetes mellitus were directly connected to hyperlipidemia. Gender, BMI, and physical activity were the parents of hyperlipidemia, that is, they were related to the occurrence of hyperlipidemia. Table 3 shows the conditional probability table of hyperlipidemia. It can be seen from the table that men who are physically inactive and obese have the highest detection risk of hyperlipidemia, with a detection rate of 75.273%. Hypertension and diabetes mellitus were the child nodes of hyperlipidemia, that is, hyperlipidemia was associated with the occurrence of hypertension and diabetes mellitus. Smoking, central obesity, daily average salt intake, and average daily oil intake were indirectly linked to hyperlipidemia.

**Table 3:** Hyperlipidemia Reasoning Condition Probability Table

activity	gender	BMI	Hyperlipdemia(%)	
			NO	YES
Insufficient	male	<18.5	64.581	35.149
Insufficient	male	18.5~	57.515	42.485
Insufficient	male	24.0~	37.926	62.074
Insufficient	male	28.0~	24.727	75.273
Insufficient	female	<18.5	69.397	30.603
Insufficient	female	18.5~	57.664	42.336
Insufficient	female	24.0~	52.28	41.72
Insufficient	female	28.0~	37.888	62.112
Normal	male	<18.5	94.811	5.189
Normal	male	18.5~	61.095	38.905
Normal	male	24.0~	43.688	56.312
Normal	male	28.0~	29.294	70.706
Normal	female	<18.5	82.164	17.836
Normal	female	18.5~	70.408	29.592
Normal	female	24.0~	52.875	47.125
Normal	female	28.0~	43.453	56.547
Sufficient	male	<18.5	52.358	47.642
Sufficient	male	18.5~	66.463	33.537
Sufficient	male	24.0~	43.642	56.358
Sufficient	male	28.0~	25.059	74.941
Sufficient	female	<18.5	62.195	37.805
Sufficient	female	18.5~	64.173	35.827
Sufficient	female	24.0~	55.612	44.388
Sufficient	female	28.0~	48.239	51.761

#### Risk reasoning for hyperlipidemia

**Fig. 5:**
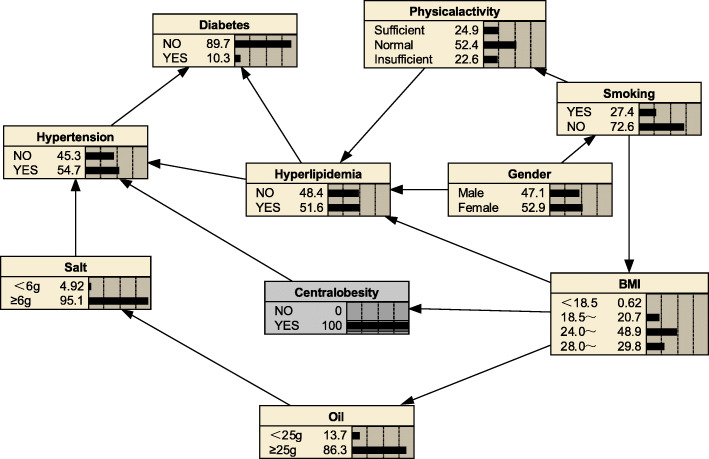
Risk reasoning for hyperlipidemia during obesity

**Fig. 6:**
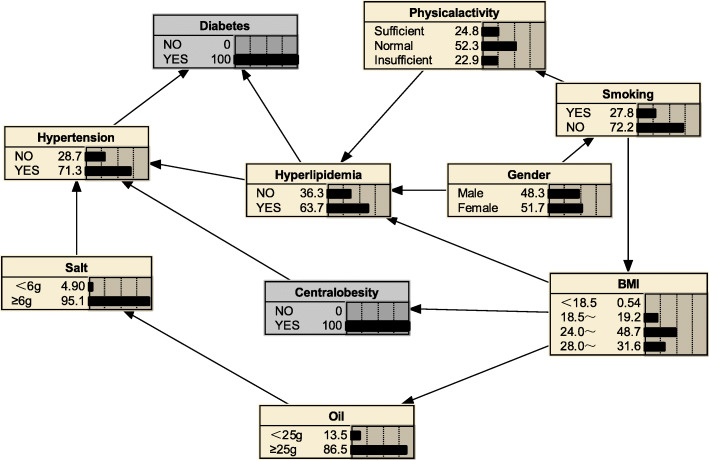
Risk reasoning for hyperlipidemia in obesity and diabetes mellitus

**Fig. 7:**
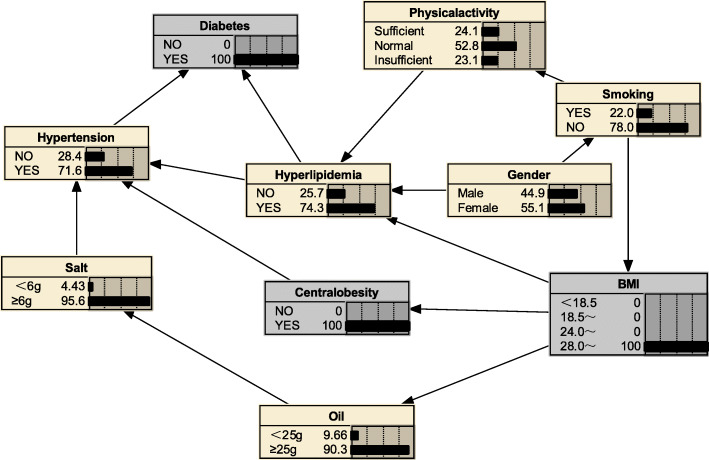
Risk reasoning for hyperlipidemia in obesity, diabetes mellitus, and central obesity

**Fig. 8:**
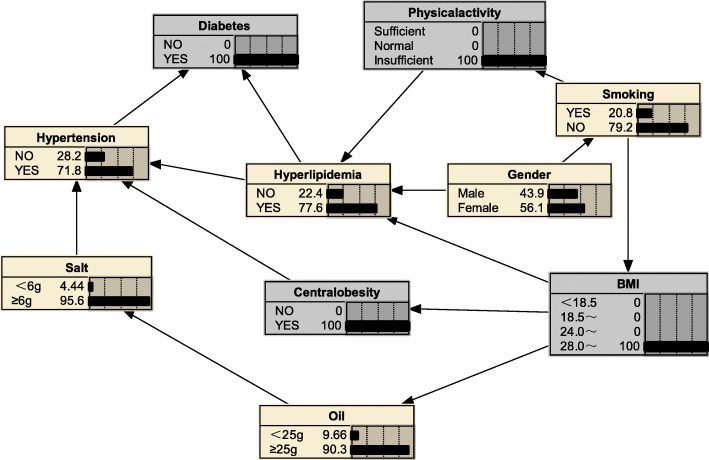
Risk reasoning for hyperlipidemia in obesity, diabetes mellitus, central obesity and lack of exercise

We can also use BNs to predict the probability of suffering from hyperlipidemia by predicting the probability of unknown nodes using information from known nodes. Figure 5 shows that if a person was judged to be central obesity based on waist circumference, the probability of developing hyperlipidemia was 0.516, i.e. *P*_*(hyperlipidemia | central obesity*)_ = 0.516; if the individual also had diabetes mellitus, the likelihood of developing hyperlipidemia increased to *P*_*(hyperlipidemia | central obesity, diabetes mellitus)*_ = 0.637(Fig. 6); if the individual’s BMI value was ≥28.0 kg/m^2^, the probability of developing hyperlipidemia increased to *P*_*(hyperlipidemia|obesity, diabetes mellitus, central obesity)*_ = 0.743(Fig. 7); if the individual was still lacking exercise, the likelihood of developing hyperlipidemia increased to *P*_*(hyperlipidemia | lack of exercise, central obesity, obesity, diabetes mellitus)*_ = 0.776(Fig. 8).

#### Correlation reasoning between other factors

The BNs of Figs. S1 and S2 can also describe the the interrelationships between the factors related to hyperlipidemia, such as the association between central obesity and BMI. When someone was obese, the likelihood of central obesity increased from 57.0 to 97.0% (Additional file 2: Fig. S1). The trend chi-square test between BMI and central obesity also showed that the higher the BMI, the higher the detection rate of central obesity, and the results supported the conclusion of the BNs(Additional file 2:Table S5); From the reasoning in Fig. S2 (Additional file 2: Fig. S2), it could be known that the average daily oil intake was indirectly related to hypertension. When the average daily oil intake was 25 g or more, the probability of developing hypertension increased by 0.3% (45.6–45.3%). Hyperlipidemia, which is directly associated with hypertension, also increased from 46.3 to 46.8%. The reasoning of the BNs was in line with medical theory. The partial regression coefficient of daily average oil intake in logistic regression was − 0.357 and OR was 0.7. It is believed that the risk of developing hyperlipidemia was reduced by 30.0% when the average daily oil intake was 25 g or more, which was against the medical theory. This may be caused by the collinearity among explanatory variables.

## Discussion

The increasing prevalence of hyperlipidemia had become a worldwide public health problem. We found the detection rate of hyperlipidemia in Shanxi Province of China in 2015 was 46.3%, which is significantly higher than the nationally reported prevalence of dyslipidemia [5] (41.9%). Numerous studies have shown that hyperlipidaemia can cause a variety of diseases and is closely associated with the occurrence of stroke, myocardial infarction, cardiac complications, diabetes mellitus, hypertension, fatty liver, pancreatitis and is one of the main factors in the formation of coronary heart disease [33–36]. In addition, hyperlipidemia generally has no obvious clinical symptoms, so it is not easy to attract people’s attention, and it is often found during physical examinations or other inspections. Although there are no obvious symptoms of dyslipidemia, it may cause disability or death once it develops. Therefore, it is particularly important to find out the risk factors that affect the occurrence of hyperlipidemia and understand the interrelationship between related factors, so as to provide early intervention for the occurrence and development of hyperlipidemia.

Considering that the traditional Logistic regression analysis method cannot show the complex linkage effects between disease-related factors, and cannot distinguish between direct and indirect factors related to hyperlipidemia; while BNs can present complex network relationships between variables, but the more variables and the more complex the network, the larger the sample size required. Hence, we will perform BNs modeling on the basis of feature selection by Logistic regression. That is, firstly, multivariate Logistic regression analysis is used to initially screen the factors related to hyperlipemia, and the selection boundary value can be more relaxed. Then use BN to construct the network of the selected variables, and finally determine the influence of these factors on hyperlipidemia, and provide a comprehensive strategy for effectively reducing the incidence of hyperlipidemia. In the BNM, considering some of the shortcomings of the MMHC hybrid algorithm, the inter.iamb-tabu hybrid algorithm with the best modeling performance was introduced to explore the related factors of hyperlipidemia(Table 1).

In this study, the BNs constructed by the inter.iamb-tabu hybrid algorithm recognizes that gender, BMI, and physical activity are the parents of hyperlipidemia, that is, they are related to the occurrence of hyperlipidemia. For physically inactive and obese men, the risk of hyperlipidaemia was higher with a conditional probability of 75.273%; diabetes mellitus and hypertension were the child nodes of hyperlipidemia, i.e. hyperlipidemia was associated with the development of diabetes mellitus and hypertension. Previous studies have shown that even after adjusting for other relevant variables, diabetes mellitus and hypertension are still associated with dyslipidemia [37, 38], consistent with the results of this article. It is recommended that men should exercise moderately and control their body weight to reduce the risk of hyperlipidemia. On this basis, they also reduce the risk of diabetes mellitus and high blood pressure. The BNs also suggests that smoking, daily salt intake, daily oil intake, and central obesity are mainly related to the occurrence of hypertension and because hyperlipidemia is related to the occurrence of hypertension. Therefore, smoking, daily salt intake, average daily oil intake, and central obesity are indirectly related to hyperlipidemia, which is in line with the theory of disease development. Logistic regression can only suggest that these factors are related to the occurrence of hyperlipidemia, and cannot distinguish whether the factors are directly or indirectly related to the disease, the results are relatively shallow.

The BNs also can be used for disease risk prediction, called BNs reasoning, to infer the probability of another unknown node based on the state of a known node, and to determine the risk of hyperlipidemia. If a person is determined to be central obesity based on waist circumference, the probability of developing hyperlipidemia is 0.516; if the individual also has diabetes mellitus, the likelihood of developing hyperlipidemia becomes 0.637, an increased risk of 12.1%; if also has a BMI ≥ 28, the probability of developing hyperlipidaemia is 0.743, a further increased risk of 10.6%; if an individual remains physically deficient, the probability of developing the probability of hyperlipidaemia increases to 0.776. The reasoning process has a sequential nature, and the risk intensity of the disease is evaluated according to the change of the conditional probability of a certain factor, indicating its value in the prevention work; the inference works even if there is some missing information; furthermore, we see from the risk inference process that changes in the level of a factor in the network are followed by different levels of conditional the probabilities change accordingly, reflecting the overall connection of things and providing a more effective, comprehensive description of the strength of the relationship between each factor and the disease. Thus, BNs are a good complement to traditional Logistic regression analysis in terms of explaining the complex links between disease-related factors.

Notably, logistic regression analysis showed a 30% reduction in the risk of hyperlipidaemia in the high oil intake group, which clearly defies medical theory. The reason maybe relate to the obvious correlation between various life behavior habits, which does not meet the independent assumptions between variables in logistic regression, resulting in the opposite sign of the regression coefficients; the BNs shows that the average daily oil intake is indirectly related to hypertension. When the average daily oil intake is 25 g or more, the possibility of developing hypertension increases by 0.3%, and hyperlipidaemia, which is directly related to hypertension, increased from 46.3 to 46.8%, precisely because BNs are able to reflect the overall association between variables and are suitable for the study of non-independent complex network relationships between variables. Its reasoning is more in line with medical theory.

In summary, the three hybrid algorithms for constructing BNs proposed by this topic have certain innovations, and the inter.iamb-tabu algorithm used to construct the BNs of hyperlipidemia will provide a more reasonable method for the study of chronic disease related factors.

Of course, there are still some shortcomings in this study: (1) We only use one benchmark network to study the performance of the algorithm and there is a need to expand the number of benchmark networks; (2) There is a lack of family history in the investigation of risk factors for hyperlipidemia, and there are certain limitations in the indicators of poor lifestyle behaviour; (3) This study uses ross-sectional survey data to construct a BNs. The direction of the edge was determined by data-driven. Further validation is needed in order to determine causality.

## Conclusion

The detection rate of hyperlipidemia in Shanxi Province in 2015 was 46.3%, which remains a substantially high incidence. We propose a BNs model that includes basic demographic characteristics, physical conditions and living habits risk factors. It can not only capture the complex network connection between different predictors, but also infers the individual probability of developing hyperlipidemia. The BNs model will facilitate physicians implement preventive and other protective strategies for people at higher risk of hyperlipidemia to reduce the incidence of hyperlipidemia.

In the next study, we will try to use multiple benchmark networks to jointly evaluate the performance of the algorithm and further verify the accuracy of algorithm modeling. Besides, we will further collect more variable information to construct a more complete BNs model of the factors associated with hyperlipidemia. In addition, we will try to further research and analysis of “Dynamic” Bayesian networks methods, taking into account the influence of time factor, and apply it to the medical field.

## Supplementary Information

**Additional file 1.** Data sources and definitions.

**Additional file 2: Table S1** Detection rate of hyperlipidemia with basic characteristics of different populations. **Table S2.** Comparison of detection rates and differences of hyperlipidemia in different lifestyles and eating habits. **Table S3.** Comparison of the detection rates and differences of hyperlipidemia in different physiological conditions and comorbidities. **Table S4.** Variables and their assignments. **Table S5.** Different BMI central type obesity detection rate. **Fig. S1.** Risk reasoning of BMI and central obesity. **Fig. S2.** Risk reasoning for hyperlipidemia and average daily oil intake.

**Additional file 3.** R codes for building the BNs.

## Data Availability

The data that support the findings of this study are available from the corresponding author upon reasonable request.
