# Supplementary material for: Application of a novel hybrid algorithm of Bayesian network in the study of hyperlipidemia related factors: a cross-sectional study
Source: BMC Public Health. 2021 Jul 12;21:1375. doi: 10.1186/s12889-021-11412-5 (PMC8273956; doi:10.1186/s12889-021-11412-5)
Supplement: Supplementary file 1 — Additional file 1. Data sources and definitions. [file 12889_2021_11412_MOESM1_ESM.docx]

**Instance dataset**

Using questionnaires, body measurements, laboratory tests, etc. to obtain the required sample data. The survey content mainly includes some basic demographic factors (such as region, gender, age, education level, occupation), lifestyle (such as smoking, drinking, dietary structure, physical activity), past medical history (such as diabetes, high blood pressure). Body measurements primarily measure height, weight, waist circumference, and blood pressure; laboratory tests include blood glucose, blood lipids, and glycated hemoglobin.

**Survey content and method:** (1) Questionnaire survey: Before collecting the data, all participants received a written informed consent. After signing the informed consent form, all participants were asked to fill a chronic disease questionnaire developed by the Chinese Center for Disease Control and Prevention (CDC). Uniformly trained investigators conducted direct face-to-face questionnaire interviews. The questionnaire included the following information: general demographic characteristics (such as age, gender, region, occupation, and education level), lifestyles (such as eating habits, drinking, smoking, and physical activity) and past medical history (such as hyperlipidemia and hypertension). (2) Anthropometric measures: Body measurement mainly involves height, weight, waist circumference and blood pressure. When measuring height and weight, participants is are required to take off his shoes, hat and coat. The measuring tools are a height meter with an accuracy of 0.1 cm and an electronic scale with 0.1 g. Waist measurement uses a waist ruler with an accuracy of 0.1 cm. Repeat the measurement twice. After ensuring that the error of the two measurements is less than 2 cm, the second measurement shall prevail. The blood pressure was measured when participants are sitting and resting for 5 minutes. Third consecutive blood pressure (BP) readings were taken by an electronic sphygmomanometer (OMRON HEM-7071 or HEM-770A), with an accuracy of 1 mmHg; finally, take the average of the three blood pressure measurements. (3) Laboratory assays: Detection indicators include blood sugar, blood lipids, glycosylated hemoglobin, etc. The samples for blood glucose testing should be stored in refrigerator at 2～8℃ and sent to the local designated laboratory for testing within 48 hours; other blood samples need to be stored at a low temperature of ﹣60℃ to ﹣80℃. In areas where there is no ultra-low temperature storage equipment, they should be stored at ≤﹣20℃ and sent to the nationally designated medical inspection agency for unified determination within one month.

**Definitions**

Dyslipidemia was defined according to criteria of the 2007 Chinese Guidelines on Prevention and Treatment of Dyslipidemia in Adults. High total cholesterol (TC) was defined as ≥ 6.22 mmol/L and hypertriglyceridemia as serum triglycerides level ≥ 2.26 mmol/L. Low high-density lipoprotein cholesterol (HDL-C) was defined as serum HDL-C <1.04 mmol/L. High low-density lipoprotein cholesterol (LDL-C) was defined as serum LDL-C ≥ 4.14 mmol/L. Hyperlipidemia is defined as one or more of the following abnormal lipid characteristics: elevated concentration of total cholesterol (TC), low-density lipoprotein cholesterol (LDL-C), triglycerides (TG) or decreased level of high-density lipoprotein cholesterol (HDL-C) ([1](#_ENREF_1)). Hypertension, according to Guidance on Prevention and Control of Hypertension in Chinese Residents, hypertension was defined as individuals with an average measured systolic blood pressure ≥ 140 mmHg and/or diastolic blood pressure ≥ 90 mmHg, or who reported having been diagnosed with hypertension or receiving BP-lowering treatment([2](#_ENREF_2)). Participants who reported smoking ≥ 1 cigarette a day for the previous 6 months were defined as smokers. Drinking refers to drinking alcohol at least 1 times a week, with an alcohol intake of 50 g or more for 6 consecutive months; Body weight was categorized as normal weight (body mass index (BMI) ≥ 18.5 kg/m2 and < 24 kg/m2), overweight (BMI ≥ 24 kg/m2 and < 28 kg/m2), and obese (BMI ≥ 28 kg/m2)([3](#_ENREF_3)). Central obesity refers to waist circumference ≥ 85 cm for males and ≥ 80 cm for female([4](#_ENREF_4)). Physical activity is classified into insufficient physical activity, physical activity reaches the standard, and adequate physical activity according to the upper quartile and lower quartile of metabolic equivalents.

**References**

1. Huang Y, Gao L, Xie X, Tan S. Epidemiology of dyslipidemia in Chinese adults: meta-analysis of prevalence, awareness, treatment, and control. Population Health Metrics. 2014.

2. M H, Y W, L Y, J Y, Y M, B H, et al. Prevalence, awareness, treatment, and control of hypertension and associated risk factors among adults in Xi'an, China: A cross-sectional study. Medicine. 2016;95(34):e4709.

3. X L, Y L, L L, L Z, Y R, H Z, et al. Prevalence, awareness, treatment, control of type 2 diabetes mellitus and risk factors in Chinese rural population: the RuralDiab study. Scientific reports. 2016;6(undefined):31426.

4. X H, Z Z, J L, W S, Y C, Y L, et al. Prevalence, awareness, treatment, and control of hypertension among China's Sichuan Tibetan population: A cross-sectional study. Clinical and experimental hypertension (New York, NY : 1993). 2016;38(5):457-63.
