# Supplementary material for: Application of a novel hybrid algorithm of Bayesian network in the study of hyperlipidemia related factors: a cross-sectional study
Source: BMC Public Health. 2021 Jul 12;21:1375. doi: 10.1186/s12889-021-11412-5 (PMC8273956; doi:10.1186/s12889-021-11412-5)
Supplement: Supplementary file 2 — Additional file 2: Table S1 Detection rate of hyperlipidemia with basic characteristics of different populations. Table S2. Comparison of detection rates and differences of hyperlipidemia in different lifestyles and eating habits. Table S3. Comparison of the detection rates and differences of hyperlipidemia in different physiological conditions and comorbidities. Table S4. Variables and their assignments. Table S5. Different BMI central type obesity detection rate. Fig. S1. Risk reasoning of BMI and central obesity. Fig. S2. Risk reasoning for hyperlipidemia and average daily oil intake. [file 12889_2021_11412_MOESM2_ESM.docx]

| Factors | groups | num | HLP | rates(%) | χ^2^ | *P-value* |
| --- | --- | --- | --- | --- | --- | --- |
| gender | male | 2236 | 1116 | 49.9 | 23.116 | ＜0.001 |
|  | female | 2331 | 998 | 42.8 |  |  |
| age | ＜40 | 549 | 121 | 48.3 | 1.116 | 0.572 |
|  | 40～ | 2202 | 273 | 46.3 |  |  |
|  | 60～ | 1816 | 945 | 45.7 |  |  |
| marital status | spinsterhood | 160 | 79 | 49.4 | 1.360 | 0.507 |
|  | married | 4178 | 1923 | 46.0 |  |  |
|  | discoverture | 112 | 112 | 48.9 |  |  |
| Cultural level | Below high school | 3692 | 1720 | 46.6 | 0.691 | 0.406 |
|  | High school and above | 875 | 394 | 46.3 |  |  |

Supplementary Table S1 Detection rate of hyperlipidemia with basic characteristics of different populations

| factors | groups | Num | HLP | rates(%) | χ^2^ | *P-value* |
| --- | --- | --- | --- | --- | --- | --- |
| smoking | NO | 3184 | 1425 | 44.8 | 9.946 | 0.002 |
|  | YES | 1383 | 689 | 49.8 |  |  |
| drinking | never | 4247 | 1950 | 45.9 | 10.517 | 0.015 |
|  | everyday | 129 | 66 | 51.2 |  |  |
|  | regularly | 151 | 70 | 46.4 |  |  |
|  | occasionally | 40 | 28 | 70.0 |  |  |
| Physical activity | Insufficient | 1032 | 518 | 50.2 | 8.366 | 0.015 |
|  | normal | 2381 | 1081 | 45.4 |  |  |
|  | sufficient | 1154 | 515 | 44.6 |  |  |
| daily average salt intake | ＜6g | 238 | 100 | 42.0 | 1.843 | 0.182 |
|  | ≥6g | 4329 | 2014 | 46.5 |  |  |
| daily average oil intake | ＜25g | 712 | 349 | 49.0 | 2.525 | 0.061 |
|  | ≥25g | 3855 | 1765 | 45.8 |  |  |

Supplementary Table S2 Comparison of detection rates and differences of hyperlipidemia in different lifestyles and eating habits

| Factors | groups | Num | HLP | rates(%) | χ^2^ | *P-value* |
| --- | --- | --- | --- | --- | --- | --- |
| hypertention | NO | 2500 | 1051 | 42.0 | 40.103 | ＜0.001 |
|  | YES | 2067 | 1063 | 51.4 |  |  |
| diabetes | NO | 4134 | 1856 | 44.9 | 34.012 | ＜0.001 |
|  | YES | 433 | 258 | 59.6 |  |  |
| Central obesity | NO | 1966 | 684 | 34.8 | 183.533 | ＜0.001 |
|  | YES | 2601 | 1430 | 55.0 |  |  |
| BMI | ＜18.5 | 127 | 34 | 26.8 | 211.658 | ＜0.001 |
|  | 18.5～ | 1926 | 694 | 36.0 |  |  |
|  | 24.0～ | 1715 | 880 | 51.3 |  |  |
|  | ≥28.0 | 799 | 506 | 63.3 |  |  |

Supplementary Table S3 Comparison of the detection rates and differences of hyperlipidemia in different physiological conditions and comorbidities

| Factors | Assignment |
| --- | --- |
| Gender($x_{1}$) | male^*^=0;female=1 |
| Cultural level ($x_{2}$) | Below high school ^*^=0;High school and above =1 |
| Smoke($x_{3}$) | NO^*^=0;YES=1 |
| Drink($x_{4}$) | never^*^=1; everyday=2;regularly=3;occasionally=4 |
| Activity($x_{5}$) | insufficient^*^=1;normal=2;sufficient=3 |
| Daily average salt intake ($x_{6}$) | <6g/d^*^=0;≥6g/d=1 |
| Daily average oil intake ($x_{7}$) | <25g/d^*^=0;≥25g/d=1 |
| BMI($x_{8}$) | <18.5^*^=1;18.5~=2, 24.0~=3;28.0~=4 |
| Central obesity ($x_{9}$) | NO*=0;YES=1 |
| Hypertention($x_{10}$) | NO*=0;YES=1 |
| Diabetes($x_{11}$) | NO*=0;YES=1 |
| Hyperlipmia (y) | NO*=0;YES=1 |

^* Reference level^

Supplementary Table S4 Variables and their assignments

| BMI(kg/$m^{2}$) | Num | Central obesity | Prevalence (%) | ${{}^{2}}_{trend}$ | *P-value* |
| --- | --- | --- | --- | --- | --- |
| <18.5 | 127 | 16 | 12.6 | 1421.079 | <0.001 |
| 18.5~ | 1926 | 539 | 28.0 |  |  |
| 24.0~ | 1715 | 1271 | 74.1 |  |  |
| 28.0~ | 799 | 751 | 97.0 |  |  |

Supplementary Table S5 Different BMI central type obesity detection rate


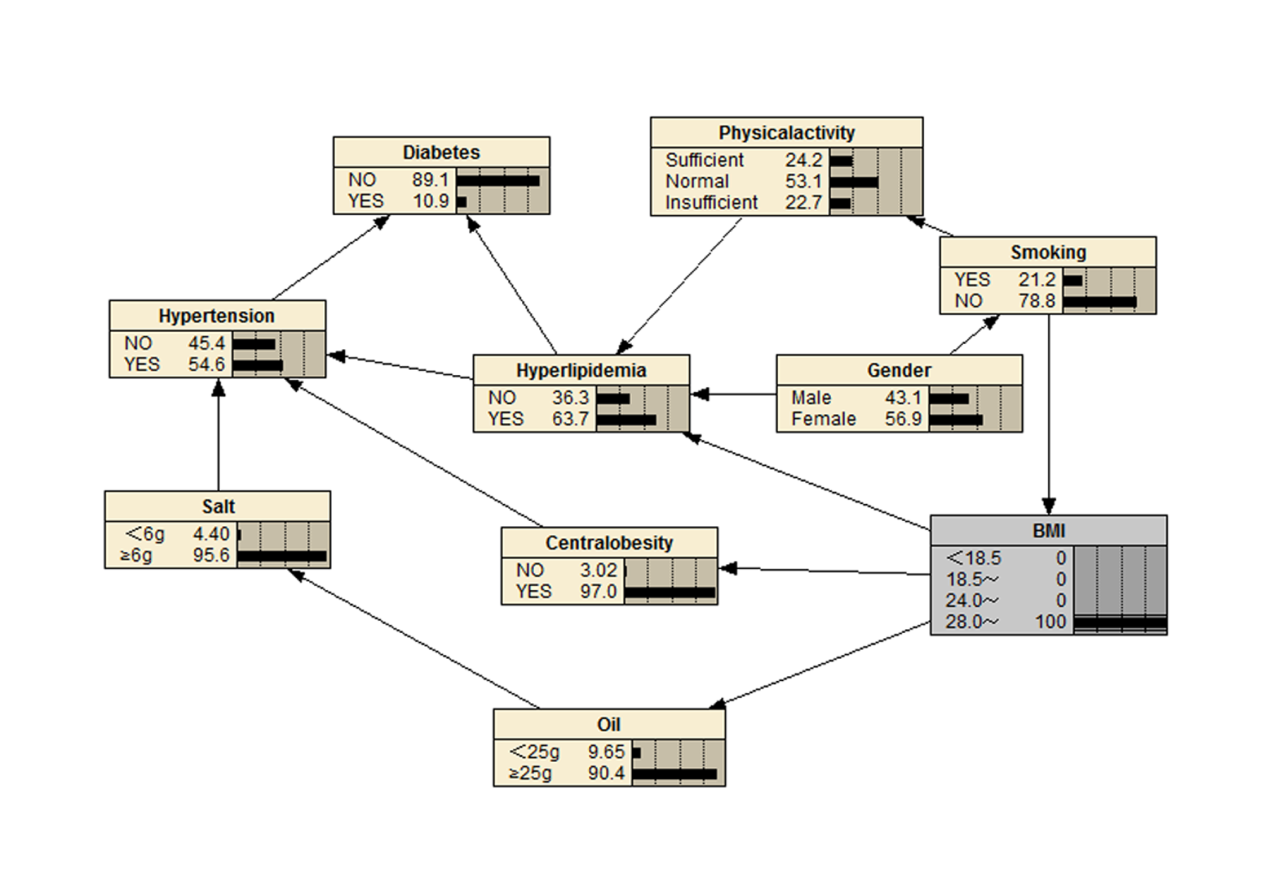
Figure S1. Risk reasoning of BMI and central obesity


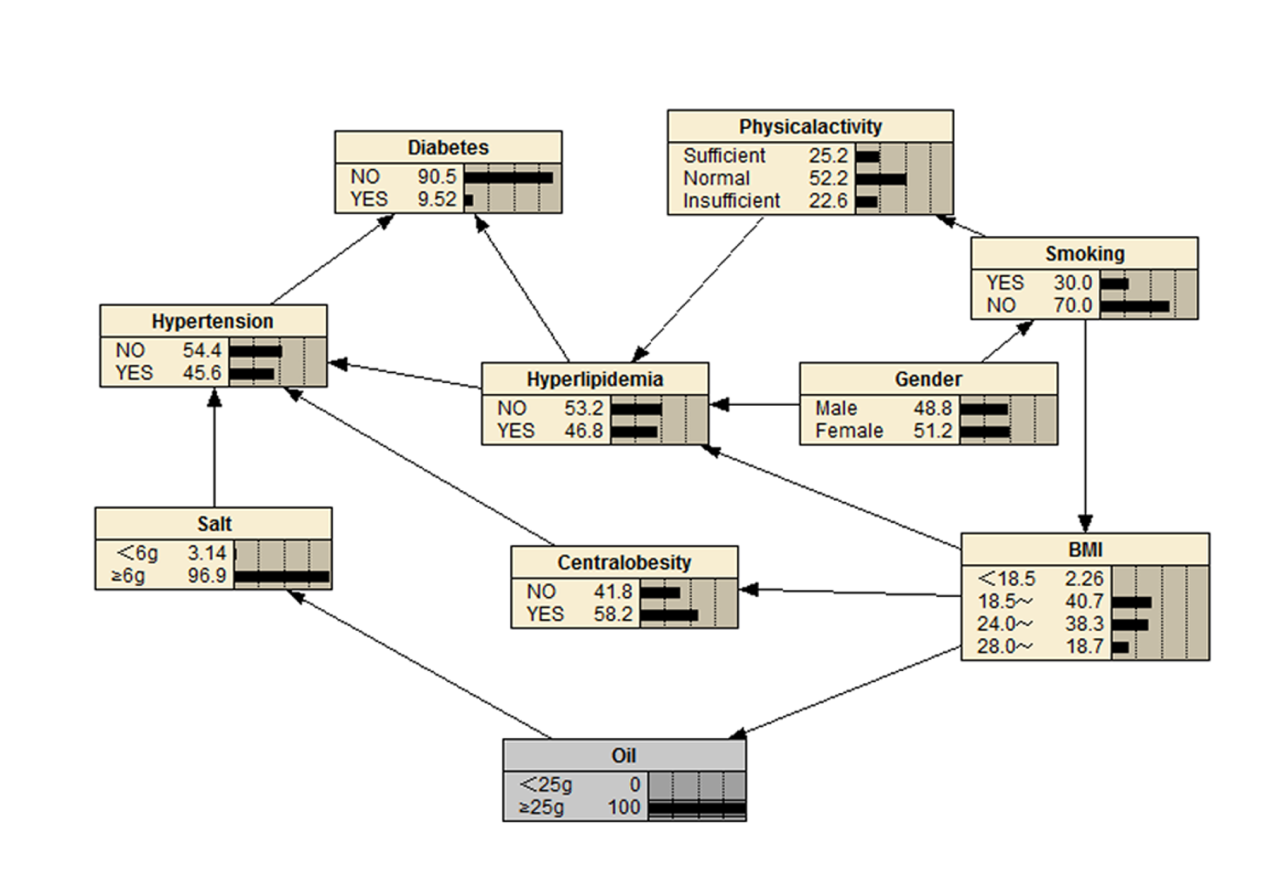


Figure S2.Risk reasoning for hyperlipidemia and average daily oil intake
