# Supplementary material for: Application of a novel hybrid algorithm of Bayesian network in the study of hyperlipidemia related factors: a cross-sectional study
Source: BMC Public Health. 2021 Jul 12;21:1375. doi: 10.1186/s12889-021-11412-5 (PMC8273956; doi:10.1186/s12889-021-11412-5)
Supplement: Supplementary file 3 — Additional file 3. R codes for building the BNs. [file 12889_2021_11412_MOESM3_ESM.docx]

**R codes for building the BNs**

**Simulating section**

mydata <- read.table("E:/ Simulating dataset/100a1.csv", header=T, sep=',' , fill=T)

library(bnlearn)

set.seed(10)

mydata=mydata[,-1]

mydata[,1]=as.factor(mydata[,1])

mydata[,2]=as.factor(mydata[,2])

mydata[,3]=as.factor(mydata[,3])

mydata[,4]=as.factor(mydata[,4])

mydata[,5]=as.factor(mydata[,5])

mydata[,6]=as.factor(mydata[,6])

mydata[,7]=as.factor(mydata[,7])

mydata[,8]=as.factor(mydata[,8])

mydata[,9]=as.factor(mydata[,9])

mydata[,10]=as.factor(mydata[,10])

mydata[,11]=as.factor(mydata[,11])

mydata[,12]=as.factor(mydata[,12])

mydata[,13]=as.factor(mydata[,13])

mydata[,14]=as.factor(mydata[,14])

mydata[,15]=as.factor(mydata[,15])

mydata[,16]=as.factor(mydata[,16])

mydata[,17]=as.factor(mydata[,17])

mydata[,18]=as.factor(mydata[,18])

mydata[,19]=as.factor(mydata[,19])

mydata[,20]=as.factor(mydata[,20])

mydata[,21]=as.factor(mydata[,21])

#MMHC#

dag=mmhc(mydata,whitelist =NULL, blacklist =NULL, restrict.args = list(),

maximize.args = list(), debug = FALSE)

plot(dag,radius=200,arrow=30)

#Fast.iamb-Tabu#

dag=rsmax2(mydata, whitelist = NULL, blacklist = NULL, restrict = "fast.iamb",

maximize = "tabu", restrict.args = list(), maximize.args = list(), debug = FALSE)

plot(dag,radius=200, arrow=30)

#Inter.iamb-Tabu#

dag=rsmax2(mydata, whitelist = NULL, blacklist = NULL, restrict = "inter.iamb",

maximize = "tabu", restrict.args = list(), maximize.args = list(), debug = FALSE)

plot(dag,radius=200,arrow=30)

dag=rsmax2(mydata, whitelist = NULL, blacklist = NULL, restrict = "mmpc",

maximize = "tabu", restrict.args = list(), maximize.args = list(), debug = FALSE)

plot(dag,radius=200,arrow=30)

**Instance section**

mydata <- read.table("E:/Instance data.csv", header=T,sep=',',fill=T)

library(bnlearn)

mydata[,1]=as.factor(mydata[,1])

mydata[,2]=as.factor(mydata[,2])

mydata[,3]=as.factor(mydata[,3])

mydata[,4]=as.factor(mydata[,4])

mydata[,5]=as.factor(mydata[,5])

mydata[,6]=as.factor(mydata[,6])

mydata[,7]=as.factor(mydata[,7])

mydata[,8]=as.factor(mydata[,8])

mydata[,9]=as.factor(mydata[,9])

mydata[,10]=as.factor(mydata[,10])

dag=rsmax2(mydata, whitelist = NULL, blacklist = NULL, restrict = "inter.iamb", maximize = "tabu", restrict.args = list(), maximize.args = list(), debug = FALSE)

proc.time()-ptm

plot(dag, radius=200,arrow=30)
